# Long-term apoptosis-related protein expression in the diabetic mouse ovary

**DOI:** 10.1371/journal.pone.0203268

**Published:** 2018-09-07

**Authors:** Nicolas A. Fraunhoffer, Analía Meilerman Abuelafia, Mariangel Aquino Barrientos, Karen Veronica Cimerman, María Florencia Olmos, Eduardo Chuluyan, Marcela Barrios

**Affiliations:** 1 Facultad de Ciencias de la Salud, Carrera de Medicina, Universidad Maimónides, Ciudad Autónoma de Buenos Aires, Buenos Aires, Argentina; 2 Consejo Nacional de Investigaciones Científicas y Técnicas (CONICET), Ciudad Autónoma de Buenos Aires, Buenos Aires, Argentina; 3 Centro de Estudios Farmacológicos y Botánicos-CONICET (CEFYBO), Buenos Aires, Argentina; Universite Paris-Sud, FRANCE

## Abstract

Emerging evidence has shown that oocytes from diabetic ovaries exhibit delayed maturation, mitochondrial dysfunction and meiotic defects, which are related increased apoptosis. The main objective of the present study was to analyze the apoptosis pathways activated during follicular loss at multiple time points in a diabetic mouse model. Twenty BALB/c mice were used in this study, and diabetes mellitus was induced by streptozotocin injection. Three diabetic and two control animals were sacrificed on days 15, 20, 70 and 80 posttreatment. The ovaries were then removed; one was used for follicular counting, TUNEL, immunohistochemistry and immunofluorescence, while the other was used for Western blot analysis. The proteins studied were BAX, BCL2, t-BID, FAS, FASL, active caspase 8, active caspase 9 and active caspase 3. Follicular apoptosis decreased over time, with the highest values observed at 15 days posttreatment. Granulosa cells were positive for active caspase 3, which showed constant expression levels at all time points. FAS, FASL, t-BID and active caspase 8 showed strong cytoplasmic immunostaining in the oocytes and granulosa cells of the diabetic mice, with significant increases observed at 15, 20 and 70 days posttreatment. BAX expression was slightly higher in the diabetic mouse ovaries than in the control ovaries at 15, 20 and 70 days posttreatment, whereas the highest active caspase 9 expression was at observed 20 days posttreatment. Low BCL2 protein levels were detected in the diabetic mouse ovaries at all time points. This study describes for the first time the behavior of apoptosis-related proteins in the diabetic mouse ovary and shows not only that the FAS/FASL pathway contributes to follicular loss but also that antral follicles are the most affected.

## Introduction

Diabetes mellitus (DM) is a chronic metabolic disorder affecting more than 6% of the global population [1]. Chronically high glucose levels have deleterious effects on the structure and function of various organs [2–4]. Tissue damage has been related to the increased formation of advanced glycation end products (AGEs) [5, 6] and the overproduction of reactive oxygen species (ROS) [2, 5], which can cause oxidative stress, vascular endothelial cell damage and inflammatory cytokine production [3, 7, 8]. Among women with DM, menstrual complications, such as polymenorrhea, oligomenorrhea and amenorrhea followed by ovulation failure, have been described [9, 10]. In addition, earlier menopause in women with DM has been described [11, 12]. In recent years, emerging evidence has shown that oocytes from diabetic rodents exhibit delayed maturation, abnormal cellular metabolism, mitochondrial dysfunction, meiotic defects and ovarian tissue damage [13–17]. These ovarian alterations produce a reduction in the ovarian reserve related to activation of the caspase 3 apoptosis pathway [18]. However, these studies analyzed the effects of DM on ovaries at specific time points without understanding the dynamic changes in apoptosis-related protein expression underlying the ovarian reserve reduction.

In this study, we characterized follicular loss in the diabetic mouse ovary at four time points and correlated follicular loss with the ovarian expression of BAX, BCL2, t-BID, FAS, FASL, active caspase 8 (C8A), active caspase 9 (C9A) and active caspase 3 (C3A). Here, we report that major follicular loss occurs during the early stage of DM induction and correlates with high protein levels of FAS, FASL, t-BID and C8A, suggesting that the FAS/FASL pathway contributes to follicular loss.

## Materials and methods

### Animals

In this study, 20 BALB/c female mice aged 60 days were used. Animals were allowed food ad libitum and housed in steel cages in a temperature-controlled environment (25 ± 4°C) under a 12-hr light/dark cycle. The experimental procedures were approved by the Institutional Committee on the Use and Care of Experimental Animals (CICUAE) of Universidad Maimónides, Argentina. Animal handling and sacrifice were performed by trained veterinary staff according to the Guide for Care and Use of Laboratory Animals publish by the US National Institute of Health (NIH Publication No. 85–23, revised 2011).

### Experimental protocols

Diabetes was induced by five intraperitoneal injections of streptozotocin (STZ) (Sigma-Aldrich, Inc., St. Louis, MO) (60 mg/kg STZ in 0.1 M sodium citrate buffer at pH 4.5). Only sodium citrate buffer was administered to the control animals. Blood glucose was verified 4 days after the final STZ injection using the ACCU-CHEK Performa Nano^®^ system (Roche Diagnostics, Japan). Mice with blood glucose levels of 300 mg/dl and higher were included in the diabetic group in this study. Then, all 20 mice (3 diabetics and 2 controls per day) were randomly euthanized at different time points (15, 20, 70 and 80 days posttreatment). These time points were selected to coincide with those described by several authors who have used STZ to induce DM [2, 9, 13, 19]. At each time point, blood samples were collected to evaluate the glucose levels, and the ovaries were removed and weighed. One ovary was fixed in 4% neutral-buffered paraformaldehyde (PFA) for histological analysis, immunohistochemistry, immunofluorescence and TUNEL, while the other was frozen at -80°C for Western blot analysis.

### Ovarian histology and follicular counting

PFA-fixed ovary sections (5 μm) were stained with hematoxylin and eosin using standard methods. Images were captured and analyzed using an optical microscope (BX40, Olympus Optical Corporation, Tokyo, Japan) fitted with a digital camera (390CU 3.2 Megapixel CCD Camera, Micrometrics, Spain).

Follicles were counted according to the method described by Sun et al., 2015 [20]. Three sections, 10 sections apart, from each ovary were selected for counting. Follicles were independently counted by two individuals. Follicular stages were classified as follows: primordial follicle, oocyte surrounded by a single layer of flatted granulosa cells; primary follicle, oocyte surrounded by a single layer of cuboidal granulosa cells; secondary follicle, oocyte surrounded by two layers of cuboidal granulosa cells; tertiary follicle, oocyte surrounded by more than two layers of cuboidal cells and without an antrum; and antral follicle, oocyte surrounded by multiple layers of cuboidal cells and with an antrum. Follicles with apoptotic bodies and an abnormal oocyte morphology were classified as apoptotic. Areas were measured using Micrometrics software (Micrometrics, Spain). Follicle counts were normalized to the area of each section.

### TUNEL assay

Apoptosis-associated DNA fragmentation was detected by TUNEL [21] using the In Situ Cell Death Detection Kit (Roche Diagnostics, Roche Applied Science). Mounted sections were dewaxed in xylene, rehydrated through a decreasing series of ethanol (100, 95, 70 and 50%) and PBS, permeabilized with 20 mg/ml nuclease-free Proteinase K in 10 mM Tris-HCl (Invitrogen, Life Technologies Corporation) for 30 min at 37°C, and finally blocked for endogenous peroxidase activity with 3% hydrogen peroxide in methanol for 30 min at room temperature. TUNEL was carried out following the supplier’s recommendations. The sections were incubated with the TUNEL reaction mixture (labeling solution plus the terminal transferase enzyme) for 60 min at 37°C. Images were captured and analyzed using a conventional epifluorescence microscope with UV illumination (BX40, Olympus Optical Corporation, Tokyo, Japan) and fitted with a digital camera (390CU 3.2 Megapixel CCD Camera, Micrometrics, Spain). Endometrial mouse tissue was used as a positive and negative control (S1 Fig). Positive control sections were incubated with 10 IU/ml recombinant DNase I (Invitrogen, Life Technologies Corporation) in 50 mM Tris-HCl (pH 7.5), 10 mM MgCl_2_ and 1 mg/ml BSA for 60 min at room temperature. After incubation with the enzyme, the slides were thoroughly rinsed with PBS and reprocessed for TUNEL. The negative control was processed in the same manner with omission of the terminal transferase enzyme.

### Immunohistochemistry

The samples were baked overnight at 60°C, dewaxed in xylene and hydrated through a decreasing ethanol series (100, 80, and 50%). After 10 min in PBS, heat-induced antigen retrieval was performed in a water bath at 96°C in 10 mM sodium citrate (pH 6) for 20 min. Endogenous peroxidase activity was blocked with 3% H_2_O_2_ in methanol for 30 min and washed twice in 0.01% Tween 20 in PBS buffer (PBST) (pH 7.4). Then, the sections were blocked with 1.5% blocking serum solution (VECTASTAIN Elite ABC Kit, Vector Laboratories, Burlingame, CA, USA) in PBST for 30 min. The slides were incubated with the primary antibody overnight at 4°C. The primary rabbit polyclonal antibodies used were anti-FAS (1:300; Santa Cruz Biotechnology, USA), anti-FASL (1:200; Santa Cruz Biotechnology, USA), anti-BAX (1:200; Santa Cruz Biotechnology, USA), and anti-BCL2 (1:100; Abcam, Cambridge, USA). The primary goat polyclonal antibody used was anti-cleaved BID (1:100; Santa Cruz Biotechnology, USA). The samples were then incubated for 30 min at room temperature with the goat anti-rabbit (VECTASTAIN Elite ABC Kit, Vector Laboratories, Burlingame, CA, USA) and rabbit anti-goat (VECTASTAIN Elite ABC Kit, Vector Laboratories, Burlingame, CA, USA) secondary antibodies, followed by incubation with an avidin-biotin complex (VECTASTAIN Elite ABC Kit, Vector Laboratories, Burlingame, CA, USA) for an additional 30 min at room temperature. The reaction was visualized with 3,3'-diaminobenzidine chromogen containing nickel salt (DAB Kit, Vector Laboratories, Burlingame, USA). The sections were then dehydrated and coverslipped. Images were captured and analyzed using an optical microscope (BX40, Olympus Optical Corporation, Tokyo, Japan) with a digital camera (390CU 3.2 Megapixel CCD Camera, Micrometrics, Spain).

### Immunofluorescence

Paraffin sections were dewaxed in xylene and hydrated through a decreasing ethanol series. After 10 min in PBS, heat-induced epitope retrieval was performed in a water bath at 96°C in 10 mM sodium citrate at pH 6 for 20 min. Then, the sections were blocked with blocking serum solution (Vector Laboratories, Burlingame, CA, USA) diluted in 1.5% PBST for 30 min. Slides were incubated with the primary antibody overnight at 4°C. The primary antibodies used were rabbit polyclonal anti-C3A (1:300; Abcam, Cambridge, USA), anti-C9A (1:50; Abcam, Cambridge, USA), and goat anti-C8A (1:100; Santa Cruz Biotechnology, USA). The secondary antibodies used were Alexa 555-conjugated donkey anti-rabbit (1:600; Invitrogen, Life Technologies Corporation, Carlsbad, California, USA) and Alexa 488-conjugated anti-goat (1:600; Invitrogen, Life Technologies Corporation, Carlsbad, California, USA), which were incubated with the sections for 60 min at room temperature. The slides were counterstained with mounting medium for fluorescence with DAPI (H-1200; Vector Laboratories, Burlingame, USA). Negative controls were processed in the same manner with omission of the primary antibodies. Images were captured using an optical microscope (Olympus BX40) with an attached digital camera (390CU 3.2 Megapixel CCD Camera, Micrometrics, Spain).

### Western blotting

Ovaries were homogenized (1:3 w/v) in RIPA buffer. The samples were separated by SDS-PAGE (29:1 acrylamide:bis acrylamide, Bio-Rad Laboratories, Hercules, California, USA) with a 10% running gel and 4% stacking gel, with 0.25 M Tris-glycine, pH 8.3, as the electrolyte buffer, in an electrophoresis cell (Mini-PROTEAN II Electrophoresis Cell, Bio-Rad Laboratories, Hercules, California, USA). For the Western blot analysis, proteins were electrotransferred to a polyvinylidene difluoride (PVDF) membrane (Immobilon-P, EMD Millipore Corporation, Billerica, Massachusetts, USA) at 250 mA for 2 h. For protein identification, the membranes were blocked for 1 h at room temperature with 5% powdered milk in PBS containing 0.1% Tween 20. Then, they were incubated overnight at 4°C with the rabbit polyclonal antibodies anti-FAS (1:1000; Santa Cruz Biotechnology, USA), anti-FASL (1:2000; Santa Cruz Biotechnology, USA), anti-BAX (1:2000; Santa Cruz Biotechnology, USA), anti-BCL2 (1:1000; Abcam, Cambridge, USA), anti-C3A (1:500; Abcam, Cambridge, USA), and anti-C9A (1:500; Abcam, Cambridge, USA) and the goat polyclonal antibodies anti-cleaved BID (1:1000; Santa Cruz Biotechnology, USA) and anti-C8A (1:1000; Santa Cruz Biotechnology, USA). For the immunoreaction, the membranes were incubated with horseradish peroxidase (HRP)-conjugated goat anti-rabbit IgG (1:3000 dilution, Bio-Rad Laboratories, Hercules, California, USA) or HRP-conjugated rabbit anti-goat IgG (1:3000 dilution, Bio-Rad Laboratories, Hercules, California, USA). The outcome was visualized using the ECL Plus Kit (GE Healthcare, Ltd., Amersham Place, Buckinghamshire, UK) for chemiluminescence development. To normalize the results, monoclonal anti-β-actin (1:6000 dilution, AC-15, Sigma-Aldrich, Inc., St. Louis, Missouri, USA) was used on the same membranes and revealed with HRP-conjugated goat anti-mouse IgG (1:3000 dilution, Bio-Rad Laboratories, Hercules, California, USA). The membranes were scanned using an ImageQuant 350 system (GE Healthcare Bio-Sciences AB, Uppsala, Sweden). The estimation of bands was performed using a prestained protein ladder (PageRuler, Fermentas UAB, Vilnius, Lithuania) as a molecular weight marker. The results are expressed as the optical density of each protein/optical density of β-actin.

### Statistical analysis

Data are expressed as the mean and standard deviation (SD). Differences between the control and diabetic groups were evaluated with the Wilcoxon-Mann-Whitney nonparametric test. Statistical analyses were performed using Infostat version 2013 (Grupo Infostat, FCA, Universidad Nacional de Córdoba, Argentina). Differences were considered significant when p < 0.05.

## Results

### Decrease in the number of follicles in diabetic mice

**Fig 1:**
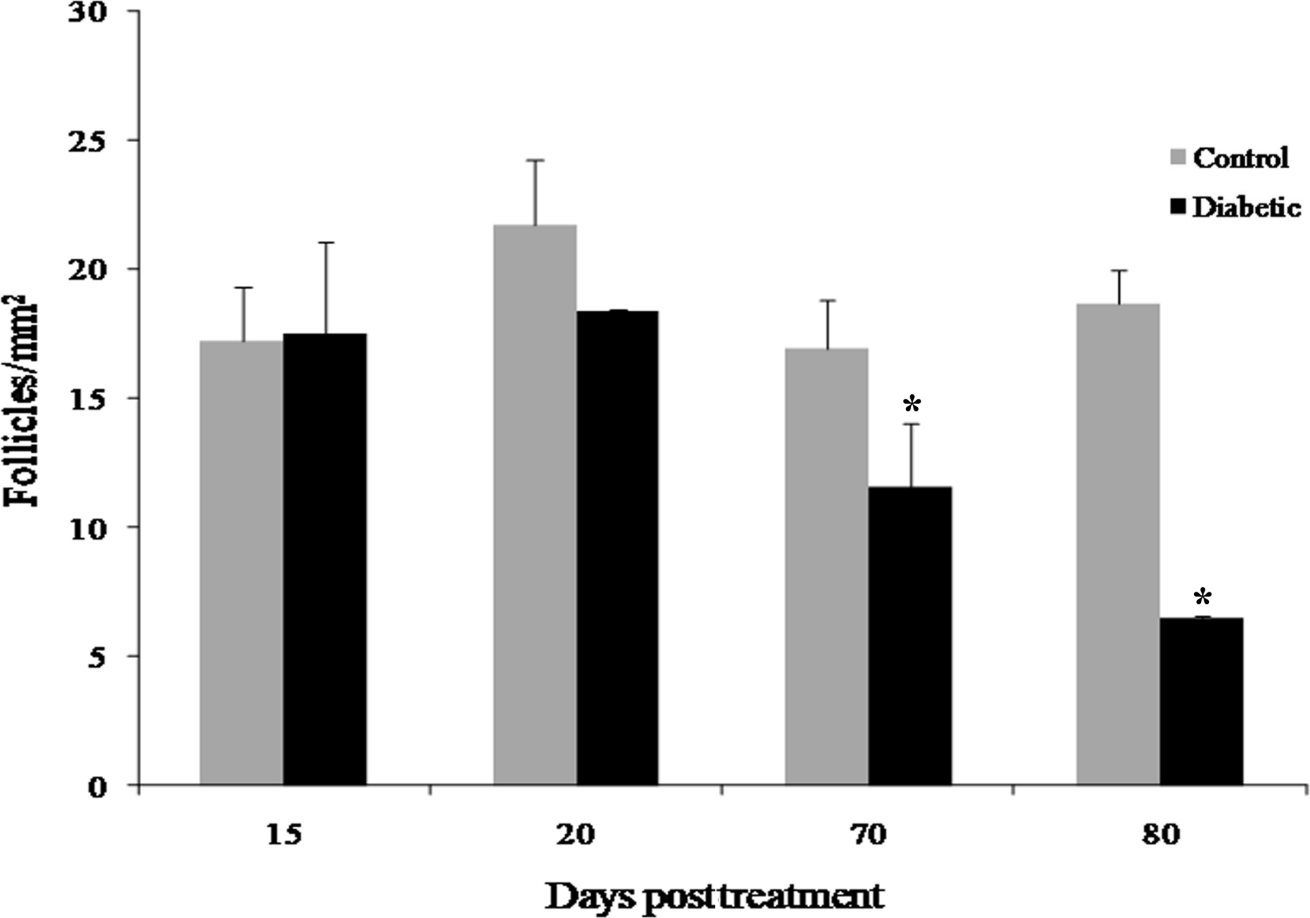
Effects of diabetes on follicular count. Total follicular count at the different time points. Bar graphs are plotted as the mean ± SD. * Significant differences, Wilcoxon-Mann-Whitney test, p < 0.05.

The diabetic mice showed markedly increased serum glucose levels at all time points compared to the control mice. In addition, the serum glucose levels were higher at the end of the study than the beginning (Table 1). The ovarian weight did not show a significant difference between the diabetic and control mice at any time point (S1 Table). However, the follicular count was significantly lower in the diabetic mice than in the control mice at 70 days posttreatment (11.54 ± 2.51 follicles/mm^2^; p = 0.0485; Fig 1; S2 Table) and 80 days posttreatment (6.46 ± 0.08 follicles/mm^2^; p = 0.0339; Fig 1; S2 Table), but not at 15 or 20 days posttreatment (Fig 1; S2 Table).

**Table 1:** Glucose levels in control and diabetic mice.

Days Posttreatment	Control^a^	Diabetic^a^
15	118.5 ± 0.71	338 ± 85.9*
20	83.5 ± 0.70	357 ± 52.9*
70	104.5 ± 3.53	399 ± 34.4*
80	129 ± 18.4	521 ± 82.3*

^a^Data are expressed as the mean ± SD.

*Statistically significant differences, p < 0.05.

### Histological alterations and increase in follicular apoptosis in diabetic mice

**Fig 2:**
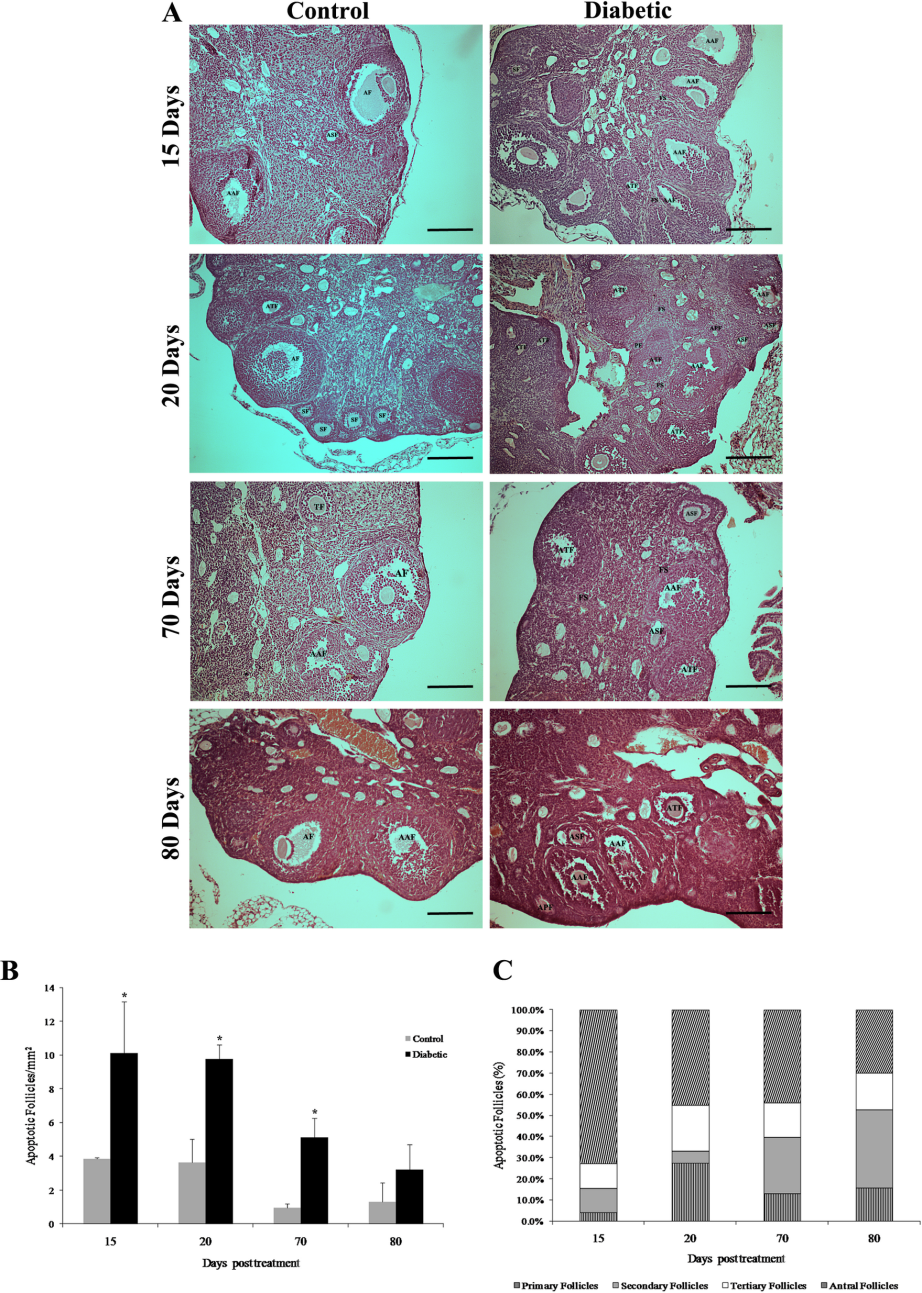
General histology and follicular apoptosis in diabetic mouse ovaries. (A) The diabetic mouse ovaries showed strong follicular degeneration associated with follicular apoptosis at different stages of maturation at all time points. (B) The number of apoptotic follicles was significantly increased in the diabetic ovaries at 15, 20 and 70 days posttreatment compared to the control group. (C) Antral follicles were the most affected at 15, 20 and 70 days posttreatment. PF, primary follicle; APF, apoptotic primary follicle; SF, secondary follicle; ASF, apoptotic secondary follicle; TF, tertiary follicle; ATF, apoptotic tertiary follicle; AF, antral follicle; AAF, apoptotic antral follicle. Bars in A represent 100 μm. Bar graphs in B are plotted as the mean ± SD. * Significant differences, Wilcoxon-Mann-Whitney test, p < 0.05.

**Fig 3:**
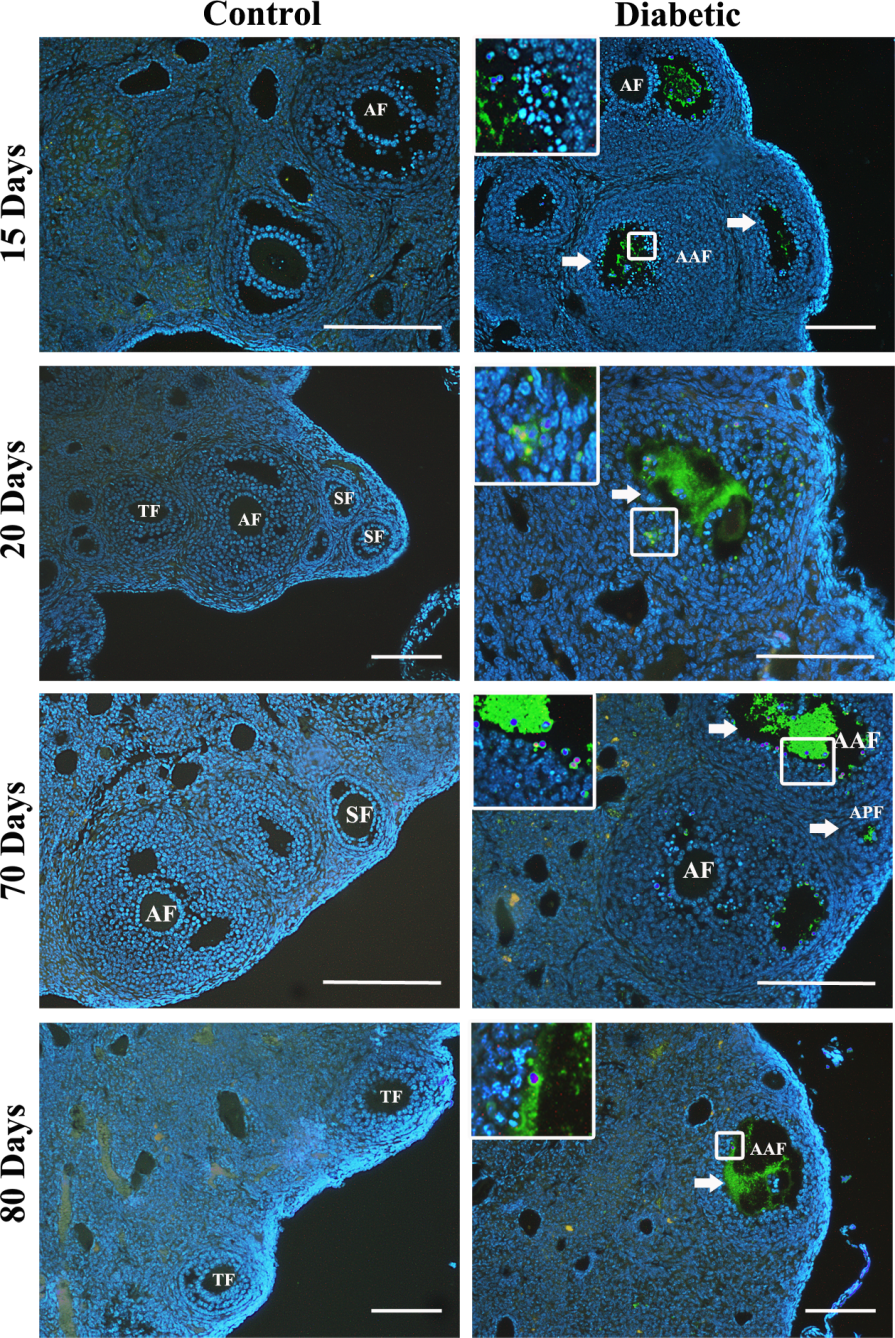
Follicular apoptosis at different time points in diabetic mouse ovaries detected by TUNEL assay. The diabetic ovaries showed positive TUNEL signals in granulosa cells (insets). Positive signal (white arrow). PF, primary follicle; APF, apoptotic primary follicle; SF, secondary follicle; ASF, apoptotic secondary follicle; TF, tertiary follicle; ATF, apoptotic tertiary follicle; AF, antral follicle; AAF, apoptotic antral follicle. Bars represent 100 μm.

In the diabetic mice, the ovarian histology revealed strong degeneration associated with follicular apoptosis, along with marked granulosa cell disorganization and abnormal oocyte morphology from the primary to antral stage (Fig 2A). In addition, granulosa cells showed strong positive TUNEL signals in the diabetic mouse ovaries (Fig 3) compared to the control mouse ovaries (Fig 3). Follicular apoptosis decreased over time, with the highest values at 15 days posttreatment (10.14 ± 3.02 apoptotic follicles/mm^2^; p = 0.0209; S3 Table) and the lowest at 80 days posttreatment (3.22 ± 1.48 apoptotic follicles/mm^2^; p = 0.0743; S3 Table) (Fig 2B). Antral follicles were the most affected, representing 70% and 40% of apoptotic follicles between 15 and 70 days posttreatment, respectively (Fig 2C). Moreover, the percentage of apoptotic primary and secondary follicles showed an increase from 15 to 80 days posttreatment (Fig 2C).

### Dynamics of apoptotic proteins in the diabetic mouse ovary

**Fig 4:**
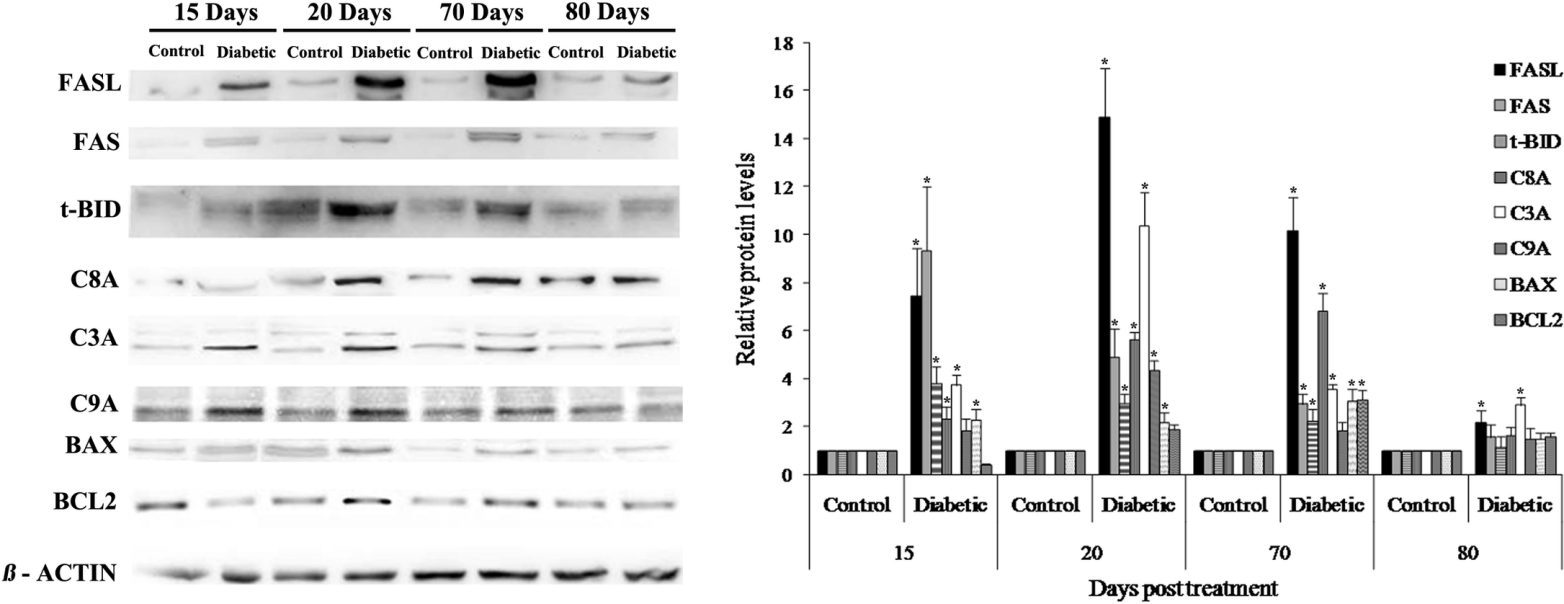
Western immunoblotting of apoptosis-related proteins. The protein levels of FAS, FASL, t-BID, C8A, C3A, and BAX were higher in the diabetic mouse ovaries than in the control mouse ovaries at 15, 20 and 70 days posttreatment. C9A expression was significantly higher in the diabetic mouse ovaries than in the control mouse ovaries at 20 days posttreatment. BCL2 showed low expression levels in the diabetic mouse ovaries. Bar graphs are plotted as the mean ± SD. * Significant differences, Wilcoxon-Mann-Whitney test, p < 0.05.

**Fig 5:**
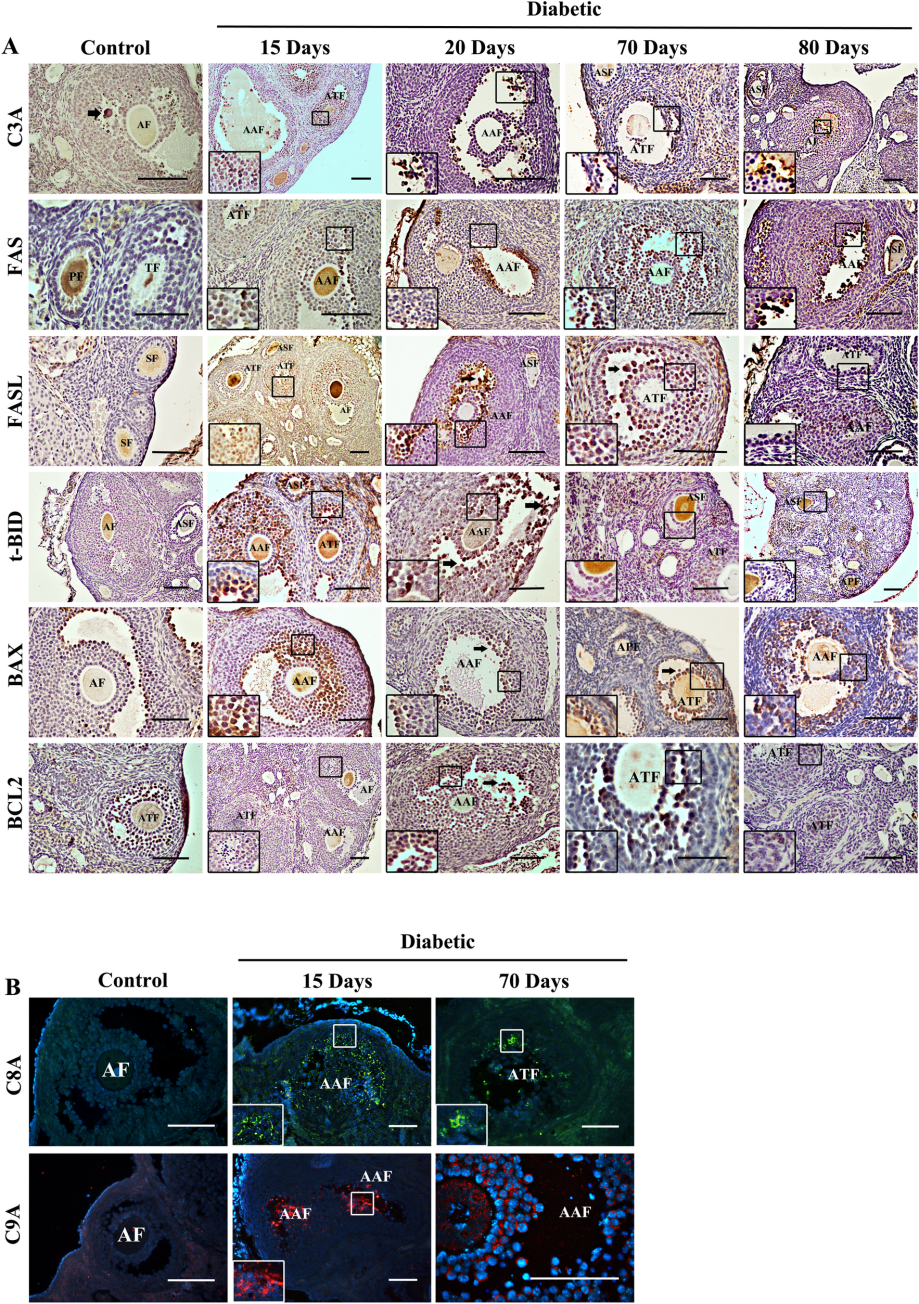
Immunohistochemical and immunofluorescence analysis of apoptosis-related proteins in diabetic mouse ovaries. (A) The diabetic mouse ovaries showed strong C3A, FAS, FASL t-BID and BAX expression in the granulosa cells and oocytes of follicles from the primary to antral stage. BCL2 showed weak immunoreactivity in the diabetic mouse ovaries compared to the control ovaries. (B) C8A and C9A expression levels were higher in the diabetic mouse ovaries than in the control mouse ovaries. Positive cells (black arrow). PF, primary follicle; APF, apoptotic primary follicle; SF, secondary follicle; ASF, apoptotic secondary follicle; TF, tertiary follicle; ATF, apoptotic tertiary follicle; AF, antral follicle; AAF, apoptotic antral follicle. Bars in A and B represent 50 μm.

To investigate the apoptotic process in diabetic mouse ovaries, proteins related to apoptosis pathways were analyzed. Ovarian C3A expression was significantly higher in the diabetic mice than in the control mice at all time points, with the highest level observed at 20 days posttreatment (Fig 4). C3A expression was strongly localized in the cytoplasm and nuclei of oocytes and granulosa cells from primary to antral follicles in the diabetic mouse ovaries at all time points, whereas C3A expression in the control mice was mainly in the granulosa cells of antral follicles (Fig 5A). The expression of FAS pathway members FAS, FASL, t-BID and C8A was significantly increased in the diabetic mouse ovaries at 15, 20 and 70 days posttreatment (Fig 4). In contrast to the control ovaries, the diabetic ovaries exhibited strong FAS and FASL expression in granulosa cells from secondary to antral follicles at all time points (Fig 5A). t-BID and C8A showed a similar immunolocalization expression pattern as FAS and FASL, with strong immunostaining in oocytes and granulosa cells (Fig 5A and 5B). C9A showed higher expression in diabetic mice than control mice at 20 days posttreatment, whereas no significant differences were detected at the other time points (Fig 4). The immunoexpression of C9A was observed mainly in oocytes and granulosa cells of antral follicles (Fig 5B). BAX expression was slightly higher in the diabetic mouse ovaries than in the control mouse ovaries at 15, 20 and 70 days posttreatment (Fig 4). The diabetic mouse ovaries showed immunostaining for BAX in the granulosa cells of follicles from the secondary to antral stage (Fig 5A). Low levels of the antiapoptotic protein BCL2 were detected in the diabetic mouse ovaries at all time points, with a significant increase at 70 days posttreatment (Fig 4). In both the diabetic and control mouse ovaries, BCL2 expression was localized predominantly in the granulosa cells of healthy and apoptotic follicles (Fig 5A).

## Discussion

Several studies have shown an association between DM and female reproductive disturbances, including amenorrhea, early menopause onset and infertility [2, 6, 9, 10, 12–18]. These alterations have been related to the injurious effects of hyperglycemia through the overproduction of AGEs and ROS, which trigger the caspase 3 apoptosis pathway [2, 4, 6, 13, 16]. However, these studies analyzed the effects of DM at a single time point, without correlating follicular loss with the expression of apoptotic proteins. For the first time, our study characterized the expression patterns of apoptosis-related proteins in the diabetic mouse ovary at multiple time points, showing that acute follicular loss occurs during the initial stages of DM. In addition, we showed that the FAS/FASL/C8A/t-BID pathway might contribute to follicular loss.

In the ovary, apoptosis is a necessary process for follicular selection during each reproductive cycle [18, 22, 23]. This selective follicular death begins with the apoptosis of granulosa and theca cells, followed by death of the enclosed oocyte. This follicular selection is triggered by activation of both the extrinsic and intrinsic apoptosis pathways [22, 23]. The intrinsic pathway is regulated by the levels of pro- and antiapoptotic members of the BCL2 superfamily, which mediate the release of cytochrome c from the mitochondria and the activation of caspase 9 [22–24]. The extrinsic pathway is initiated by the interaction of TNF superfamily ligands (FASL, TNFα and TRAIL) with their receptors (FAS, TNFR1 and DR5), which triggers the cleavage of caspase 8 and the consequent activation of BID; this activation generates t-BID, which mediates the release of cytochrome c from the mitochondria and thus causes activation of the intrinsic pathway [22–24]. Both pathways end with the activation of caspase 3, leading to cell death [22–25]. In relation to the diabetic ovary, our results confirm previous observations that demonstrated activation of the intrinsic pathway through BAX as a consequence of hyperglycemia [2, 9, 13–18, 26]. However, we observed the specific dynamics of apoptosis-related proteins, with the highest levels occurring at 15 and 20 days posttreatment in the granulose cells of antral follicles. These data suggest that a major follicular imbalance related to hyperglycemia occurs during the early stages of DM. FAS, FASL, C8A and t-BID play a central role in triggering apoptosis in the normal mammalian ovary to regulate follicular selection and luteolysis [27–29]. Here, we observed an upregulation of FAS, FASL, C8A and t-BID mainly in the granulosa cells of antral follicles, indicating that apoptosis may occur by the extrinsic pathway in the diabetic mouse ovary, whereas BAX and C9A expression might be a consequence of the cleavage of BID. However, the FAS/FASL pathway is not the only extrinsic pathway activated by hyperglycemia; previously, Chang et al. [13] reported an upregulation of the extrinsic pathway proteins TRAIL and KILLER in the diabetic mouse ovary and related this overexpression to a reduction in the gap junction protein connexin 43, which is fundamental for metabolic communication between granulosa cells and oocytes [13]. In addition, increased ovarian apoptosis mediated by FAS/FASL has been observed in polycystic ovarian rat models [30]. These observations suggest that pathologies with alterations in the glucose metabolism might activate the same apoptosis pathways related to granulosa cell metabolic communication. In relation to ROS and AGEs, it has been reported that cellular stress and a proinflammatory environment stimulate activation of the NF-κB pathway, which stimulates the expression of FAS and FAS [30–34].

Our analysis of the ovarian reserve demonstrates by direct counting that apoptosis induced by hyperglycemic toxicity significantly reduces the follicular count in diabetic mouse ovaries compared to that in control mouse ovaries at advanced disease stages. This result is contradictory to those of previous reports showing a reduction in the ovarian reserve during the early stages of DM [2, 16]. These differences might be related to these studies using anti-Müllerian hormone (AMH) as an indirect indicator of the ovarian reserve without evaluating the follicular count or the follicular stage affected by diabetic alterations. In addition, AMH is secreted by granulosa cells from the primary to antral follicular stage [35]; therefore, their observations may be related to the atretic stage of the antral follicles during the earliest pathological stage.

## Conclusions

In conclusion, the data reported here describe for the first time the behavior of apoptosis-related proteins in the diabetic ovary and show that activation of the FAS/FASL pathway in granulosa cells contributes to the mechanisms of follicular loss mainly during the early stages of DM. However, it is important to consider that the process of follicular loss may be orchestrated by the participation of multiple cell death mechanisms, such as autophagy and apoptosis, which are triggered by ROS and AGEs. Therefore, to understand the effects of DM in ovarian homeostasis, a holistic approach is necessary to evaluate the interactions among the different cell death mechanisms.

## Supporting information

S1 Fig TUNEL positive and negative control. (TIF)

S1 Table Ovarian weights. (DOCX)

S2 Table Total follicular counts. (DOCX)

S3 Table Apoptotic follicles. (DOCX)

## References

[1] Wild SH, Roglic G, Green A, Sicree R, King H. Global prevalence of diabetes: estimates for the year 2000 and projections for 2030. Diabetes care. 2004; 27(5): 1047–1053. 15111519 10.2337/diacare.27.5.1047

[2] Nayki U, Onk D, Balci G, Nayki C, Onk A, Çankaya M, et al The effect of melatonin on oxidative stress and apoptosis in experimental diabetes mellitus-related ovarian injury. Gyneco Endocrinol. 2016; 32(5): 421–426. 10.3109/09513590.2015.1126819 26743008

[3] Lee YS, Eun HS, Kim SY, Jeong JM, Seo W, Byun JS, et al Hepatic immunophenotyping for streptozotocin-induced hyperglycemia in mice. Sci Rep. 2016; 6. 10.1038/srep30656 PMC4964583 27464894

[4] Rasheed NOA, Ahmed LA, Abdallah DM, El-Sayeh BM. (2017). Nephro-toxic effects of intraperitoneally injected EGCG in diabetic mice: involvement of oxidative stress, inflammation and apoptosis. Sci Rep. 2017; 7. 10.1038/srep40617 PMC5241811 28098182

[5] Brownlee M. The pathobiology of diabetic complications: a unifying mechanism. Diabetes. 2005; 54: 1615–1625. 15919781 10.2337/diabetes.54.6.1615

[6] Merhi Z. Advanced glycation end products and their relevance in female reproduction. Hum Reprod. 2013; 29(1):135–145. 10.1093/humrep/det383 24173721

[7] Kiriakidis S, Andreakos E, Monaco C, Foxwell B, Feldmann M, Paleolog E. VEGF expression in human macrophages is NF-κB dependent: studies using adenoviruses expressing the endogenous NF-κB inhibitor IκBα and a kinase-defective form of the IκB kinase 2. J Cell Sci 2003; 116: 665–674. 12538767 10.1242/jcs.00286

[8] Karasu C. Glycoxidative stress and cardiovascular complications in experimentally induced diabetes: effects of antioxidant treatment. Open Cardiovasc Med J. 2010; 4: 240–256. 10.2174/1874192401004010240 21270942 PMC3026340

[9] Pala HG, Pala EE, Artunc Ulkumen B, Aktug H, Yavasoglu A, Korkmaz HA, Erbas O. The protective effect of granulocyte colony-stimulating factor on endometrium and ovary in a rat model of diabetes mellitus. Gynecol Obstet Invest. 2014; 78(2): 94–100.7 10.1159/000363239 25033771

[10] Zarzycki W, Zieniewicz M. Reproductive disturbances in type 1 diabetic women. Neuro Endocrinol Lett. 2005; 26(6): 733–738. 16380672

[11] Strotmeyer ES, Steenkiste AR, Foley TP, Berga SL, Dorman JS. Menstrual cycle differences between women with type 1 diabetes and women without diabetes. Diabetes Care. 2003; 26(4): 1016–1021. 12663566 10.2337/diacare.26.4.1016

[12] Schweiger BM, Snell-Bergeon JK, Roman R, McFann K, Klingensmith GJ. Menarche delay and menstrual irregularities persist in adolescents with type 1 diabetes. Reprod Biol Endocrinol. 2011; 9(1): 61. 21548955 10.1186/1477-7827-9-61 PMC3100251

[13] Chang AS, Dale AN, Moley KH. Maternal diabetes adversely affects preovulatory oocyte maturation, development, and granulosa cell apoptosis. Endocrinology. 2005; 146(5):2445–2453 10.1210/en.2004-1472 15718275

[14] Colton SA, Pieper GM, Downs SM. Altered meiotic regulation in oocytes from diabetic mice. Biol Reprod. 2002; 67(1):220–231 12080021 10.1095/biolreprod67.1.220

[15] Diamond MP, Moley KH, Pellicer A, Vaughn WK, DeCherney AH. Effects of streptozotocin- and alloxan-induced diabetes mellitus on mouse follicular and early embryo development. J Reprod Fertil. 1989; 86(1):1–10 2526873 10.1530/jrf.0.0860001

[16] Artunc-Ulkumen B, Pala HG, Pala EE, Yavasoglu A, Yigitturk G, Erbas O. Exenatide improves ovarian and endometrial injury and preserves ovarian reserve in streptozocin induced diabetic rats. Gynecol Endocrinol. 2015; 31(3):196–201. 10.3109/09513590.2014.975686 25366063

[17] Wang Q, Moley KH. Maternal diabetes and oocyte quality. Mitochondrion. 2010; 10(5): 403–410. 10.1016/j.mito.2010.03.002 20226883 PMC5028203

[18] Nayki U, Onk D, Balci G, Nayki C, Onk A, Gunay M. The effects of diabetes mellitus on ovarian injury and reserve: an experimental study. Gynecol Obstet Invest. 2015; 81(5): 424–429. 10.1159/000442287 26682912

[19] Ballester J, Muñoz MC, Domínguez J, Palomo MJ, Rivera M, Rigau T, Guinovart JJ, Rodríguez-Gil JE. Tungstate administration improves the sexual and reproductive function in female rats with streptozotocin-induced diabetes. Hum Reprod. 2007; 22(8): 2128–2135. 10.1093/humrep/dem168 17588954

[20] Sun T, Pepling ME, Diaz FJ. Lats1 deletion causes increased germ cell apoptosis and follicular cysts in mouse ovaries. Biol Reprod. 2015; 93(1):22 10.1095/biolreprod.114.118604 26040669

[21] Gavrieli Y, Sherman Y, Ben-Sasson SA. Identification of programmed cell death in situ via specific labeling of nuclear DNA fragmentation. J Cell Biol. 1992; 119(3): 493–501. 1400587 10.1083/jcb.119.3.493 PMC2289665

[22] Hussein MR. Apoptosis in the ovary: molecular mechanisms. Hum Reprod Update. 2005; 11(2): 162–178. 10.1093/humupd/dmi001 15705959

[23] Matsuda-Minehata F, Inoue N, Goto Y, Manabe N. The regulation of ovarian granulosa cell death by pro-and anti-apoptotic molecules. J Reprod Dev. 2006; 52(6): 695–705. 16926526 10.1262/jrd.18069

[24] Hutt KJ. The role of BH3-only proteins in apoptosis within the ovary. Reproduction. 2015; 149(2): R81–R89. 10.1530/REP-14-0422 25336346

[25] Boumela I, Assou S, Aouacheria A, Haouzi D, Dechaud H, et al Involvement of BCL2 family members in the regulation of human oocyte and early embryo survival and death: gene expression and beyond. *Reproduction*. 2011; 141(5): 549–561. 10.1530/REP-10-0504 21339285

[26] Moley KH, Chi MM, Knudson CM, Korsmeyer SJ, Mueckler MM. Hyperglycemia induces apoptosis in preimplantation embryos via cell death effector pathways. Nat Med. 1998; 12:1421–1424. 10.1038/4013 9846581

[27] Guan S, Guo L, Zhang T, Zhu B, Wang X, Zhang C. Effects of gonadotropin on Fas and/or FasL expression and proliferation in rat ovary. Theriogenology. 2015; 83(1): 21–29. 10.1016/j.theriogenology.2014.06.026 25294749

[28] Sapi E, Brown WD, Aschkenazi S, Lim C, Munoz A, Kacinski BM, Rutherford T, Mor G. Regulation of Fas ligand expression by estrogen in normal ovary. J Soc Gynecol Investig. 2002; 9(4): 243–250. 12113885

[29] Carambula SF, Pru JK, Lynch MP, Matikainen T, Gonçalves PBD, Flavell RA, Tilly JL, Rueda BR. Prostaglandin F2alpha-and FAS-activating antibody-induced regression of the corpus luteum involves caspase-8 and is defective in caspase-3 deficient mice. Reprod Biol Endocrinol. 2003; 1(1): 15. 12657159 10.1186/1477-7827-1-15 PMC152637

[30] Honnma H, Endo T, Henmi H, Nagasawa K, Baba T, Yamazaki K, et al Altered expression of Fas/Fas ligand/caspase 8 and membrane type 1-matrix metalloproteinase in atretic follicles within dehydroepiandrosterone-induced polycystic ovaries in rats. Apoptosis. 2006; 11(9): 1525–1533. 10.1007/s10495-006-9148-2 16820958

[31] Wang P, Xing Y, Chen C, Chen Z, Qian Z. Advanced glycation end-product (AGE) induces apoptosis in human retinal ARPE-19 cells via promoting mitochondrial dysfunction and activating the Fas-FasL signaling. Biosci Biotechnol Biochem. 2016; 80(2): 250–256. 10.1080/09168451.2015.1095065 26479732

[32] Bao W, Min D, Twigg SM, Shackel NA, Warner FJ, Yue DK, et al Monocyte CD147 is induced by advanced glycation end products and high glucose concentration: possible role in diabetic complications. Am J Physiol Cell Physiol. 2010; 299: 1212–1219. 10.1152/ajpcell.00228.2010 20810913

[33] Simon HU, Haj-Yehia A, Levi-Schaffer F. Role of reactive oxygen species (ROS) in apoptosis induction. Apoptosis. 2000; 5(5): 415–418. 11256882 10.1023/a:1009616228304

[34] Circu ML, Aw TY. Reactive oxygen species, cellular redox systems, and apoptosis. Free Radic Biol Med. 2010; 48(6): 749–762. 10.1016/j.freeradbiomed.2009.12.022 20045723 PMC2823977

[35] Fleming R, Seifer DB, Frattarelli JL, Ruman J. Assessing ovarian response: antral follicle count versus anti-Müllerian hormone. Reprod Biomed Online. 2015; 31(4): 486–496. 10.1016/j.rbmo.2015.06.015 26283017

