# Supplementary figures and images for: Long-term apoptosis-related protein expression in the diabetic mouse ovary
Source: PLoS One. 2018 Sep 7;13(9):e0203268. doi: 10.1371/journal.pone.0203268 (PMC6128485; doi:10.1371/journal.pone.0203268)

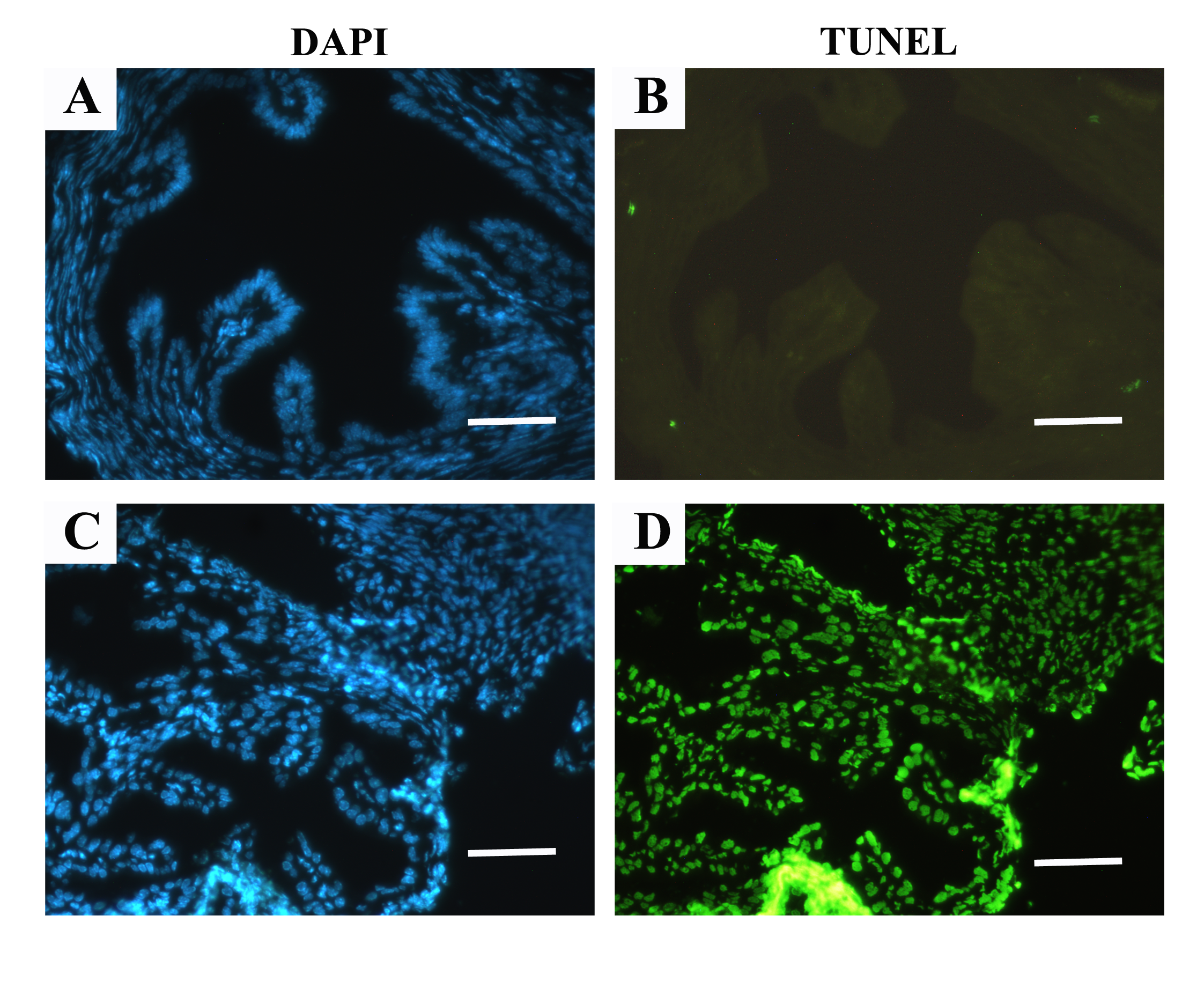

Supplement: S1 Fig — (TIF) [file pone.0203268.s001.tif]
