# Supplementary material for: Long-term apoptosis-related protein expression in the diabetic mouse ovary
Source: PLoS One. 2018 Sep 7;13(9):e0203268. doi: 10.1371/journal.pone.0203268 (PMC6128485; doi:10.1371/journal.pone.0203268)
Supplement: S1 Table — (DOCX) [file pone.0203268.s002.docx]

| **Ovary** | **Days**  **Posttreatment** | **Diabetic**  **Mean ± SD** | **Control**  **Mean ± SD** | **Statistic** | ***p*-value** |
| --- | --- | --- | --- | --- | --- |
| Right | 15 | 0.006 ± 0.002 | 0.009 ± 0.001 | W = 8.50 | 0.4000 |
|  | 20 | 0.018 ± 0.007 | 0.024 ± 0.003 | W = 8.00 | 0.4000 |
|  | 70 | 0.008 ± 0.004 | 0.014 ± 0.003 | W = 8.00 | 0.4000 |
|  | 80 | 0.008 ± 0.001 | 0.008 ± 0.001 | W = 9.00 | 0.2000 |
|  |  |  |  |  |  |
| Left | 15 | 0.012 ± 0.001 | 0.012 ± 0.001 | W = 9.00 | 0.2000 |
|  | 20 | 0.019 ± 0.006 | 0.020 ± 0.002 | W = 5.00 | 0.6000 |
|  | 70 | 0.008 ± 0.006 | 0.015 ± 0.001 | W = 8.00 | 0.4000 |
|  | 80 | 0.011 ± 0.004 | 0.012 ± 0.001 | W = 7.00 | 0.8000 |

**SUPPLEMENTAL TABLE S1**. Ovarian weights.
