# Supplementary material for: Long-term apoptosis-related protein expression in the diabetic mouse ovary
Source: PLoS One. 2018 Sep 7;13(9):e0203268. doi: 10.1371/journal.pone.0203268 (PMC6128485; doi:10.1371/journal.pone.0203268)
Supplement: S2 Table — (DOCX) [file pone.0203268.s003.docx]

| **Days**  **Posttreatment** | **Diabetic**  **Mean ± SD** | **Control**  **Mean ± SD** | **Statistic** | ***p*-value** |
| --- | --- | --- | --- | --- |
| 15 | 17.49 ± 3.55 | 17.19 ± 2.14 | W = 10.00 | 0.8273 |
| 20 | 18.36 ± 0.06 | 21.70 ± 2.56 | W = 3.00 | 0.0833 |
| 70 | 11.54 ± 2.51 | 16.94 ± 1.84 | W = 15.00 | 0.0485 |
| 80 | 6.46 ± 0.09 | 18.66 ± 1.29 | W = 18.00 | 0.0339 |

**SUPPLEMENTAL TABLE S2**. Total follicular counts
