# Supplementary material for: Long-term apoptosis-related protein expression in the diabetic mouse ovary
Source: PLoS One. 2018 Sep 7;13(9):e0203268. doi: 10.1371/journal.pone.0203268 (PMC6128485; doi:10.1371/journal.pone.0203268)
Supplement: S3 Table — (DOCX) [file pone.0203268.s004.docx]

| **Days**  **Posttreatment** | **Diabetic**  **Mean ± SD** | **Control**  **Mean ± SD** | **Statistic** | ***p*-value** |
| --- | --- | --- | --- | --- |
| 15 | 10.14 ± 3.02 | 3.86 ± 0.072 | W = 10.00 | 0.0209 |
| 20 | 9.76 ± 0.84 | 3.64 ± 1.38 | W = 18.00 | 0.0339 |
| 70 | 5.12 ± 1.14 | 0.96 ± 0.24 | W = 10.00 | 0.0209 |
| 80 | 3.22 ± 1.48 | 1.31 ± 1.13 | W = 9.00 | 0.0743 |

**SUPPLEMENTAL TABLE S3**. Apoptotic follicles.
